# Effect of natural compounds on NRF2/KEAP1 signaling in periodontitis: a potential use to prevent age-related disorders

**DOI:** 10.1007/s11033-025-10878-5

**Published:** 2025-07-30

**Authors:** Giovanni Tossetta, Sonia Fantone, Fabiola Olivieri, Roberta Mazzucchelli, Lucrezia Togni, Andrea Santarelli, Daniela Marzioni, Maria Rita Rippo

**Affiliations:** 1 Department of Experimental and Clinical Medicine, Università Politecnica delle Marche, Ancona, 60126 Italy; 2 Department of Biomedical Sciences and Public Health, Section of Pathological Anatomy, Università Politecnica delle Marche, United Hospitals, Ancona, Italy; 3 Scientific Direction, IRCCS INRCA, Ancona, 60124 Italy; 4 Department of Clinical and Molecular Sciences, DISCLIMO, Università Politecnica delle Marche, Ancona, 60126 Italy; 5 Department of Clinical Specialistic and Dental Sciences, Università Politecnica delle Marche, Ancona, 60126 Italy; 6 Dentistry Clinic, National Institute of Health and Science of Aging, IRCCS INRCA, Ancona, 60126 Italy; 7 IRCCS INRCA, Ancona, 60124 Italy

**Keywords:** Alzheimer’s disease, Nuclear factor erythroid 2-related factor 2 (NRF2), Oxidative stress, Parkinson’s disease, Periodontitis

## Abstract

40% of the population over 60 years of age is affected by periodontitis which is characterized by chronic inflammation, periodontal damage and alveolar bone resorption. The nuclear factor erythroid 2-related factor 2 (NFE2L2 or NRF2)/ Kelch-like ECH-Associated Protein 1 (KEAP1) (NRF2/KEAP1) signaling pathway plays a key role in periodontitis modulating redox balance and periodontium inflammation. However, NRF2 expression decreases in gingival tissues of severe periodontitis patients while Reactive Oxygen Species (ROS) levels are increased during periodontitis. ROS and lipopolysaccharide (LPS) produced by gram-negative bacteria favor the production of inflammatory cytokines, then causing periodontal inflammation and favoring alveolar bone loss (due to excessive osteoclast formation and activation). Periodontitis has also been associated to the development of age-related neurodegenerative diseases such as Alzheimer’s and Parkinson’s diseases since the increased cytokines levels and the bacteria themselves present in the periodontium can easily reach the brain due to their anatomical proximity. Thus, periodontitis could be considered a risk factor for the development of Alzheimer’s and Parkinson’s diseases. In this review we explored the role of NRF2/KEAP1 signaling activation in in vitro and in vivo models of periodontitis to suggest potential treatments of periodontitis and avoid/delay the development of age-related neurodegenerative diseases.

## Introduction

Periodontitis is an inflammatory disease that can damage the periodontium leading to progressive destruction of the alveolar bone supporting the teeth [1]. The main clinical features of periodontitis include periodontal tissue loss and periodontal pocketing and gingival bleeding, induced by the clinical attachment loss (CAL) and the alveolar bone loss [2]. Since periodontitis is among the major causes of edentulism and masticatory dysfunction, it has a negative impact on general health and impairs the quality of life [3, 4]. Moreover, although dental implants show beneficial effects edentulism treatment [5, 6], it has been demonstrated that patients with periodontitis have an higher risk to develop peri-implantitis [7]. According to the Global Burden of Disease, periodontitis has an overall prevalence of 61.6% [8]. Moreover, prevalence and the severity of periodontitis significantly increase with age [9], the concept of periodontitis as an inevitable consequence of aging has been challenged over the years. Periodontitis represents the cumulative effect of prolonged exposure to several risk factors such as microbial factors, smoking, systemic diseases (i.e. diabetes mellitus), poly-pharmacological therapies, and lifestyle. However, age-associated molecular alterations of immunological cells have been shown to affect their ability to carry out efficient antimicrobial functions, resulting in a dysregulation of the inflammatory response [10]. Therefore, an age‐related, rather than an age‐dependent, increased susceptibility to periodontitis in elderly is biologically plausible.

The role of oral microbiota dysbiosis in the pathogenesis of periodontitis has been highly recognized, and its progression has been linked to an excessive host response to the biofilm of the sub-gingival plaque with a consequent impairment of the homeostatic balance between the commensal microbiota and the immune and inflammatory systems of the tissues [11].

Among various microorganisms that can colonize the mouth, *Porphyromonas gingivalis*,* Tannerella forsythia* and *Aggregatibacter actinomycetemcomitans*, as well as their virulence factors such as lypopolisaccharide (LPS) and cytolitic enzymes, are the most common pathogens involved in periodontitis occurrence [12].

Neutrophils, the first defense line against bacterial infection, are the most common inflammatory cells present in periodontitis. Neutrophils produce a large amount of oxygen reactive species (ROS) in periodontal tissue and gingival sulcus during periodontitis to fight the pathogens present in the dysbiotic biofilm [13]. For this reason, neutrophils play a pivotal role in the progression of this pathology. In fact, it has been widely demonstrated that ROS such as hydroxyl radical (OH^−^), hydrogen peroxide (H_2_O_2_) and superoxide (O_2_^−^) have an important role in the onset and progression of several human pathologies including cancer [14, 15], inflammatory diseases [16, 17], and periodontitis [18, 19]. However, ROS can also be produced from fibroblasts of periodontal tissue in the presence of lipopolysaccharide (LPS) produced by gram-negative bacteria such as *P. gingivalis* [20].

Since bone homeostasis is granted by a tightly regulated balance between bone resorption (by osteoclasts) and bone formation (by osteoblasts), the pro-inflammatory context of periodontitis alters this balance. In fact, the pro-inflammatory cytokines such as tumor necrosis factor (TNF)-α, interleukin (IL)-1, and IL-6 favor an excessive osteoclast formation/activation promoting alveolar bone loss [21].

An excessive bone resorption due to an increased number or activity of osteoclasts is the cause of several bone diseases including rheumatoid arthritis, osteoporosis, peri-implantitis and periodontitis [21–25]. For this reason, the regulation of osteoclasts formation and function has important therapeutic implications.

The receptor activator of nuclear factor-κB ligand (RANKL), generally produced by osteoblasts, plays a key role in osteoclast differentiation and thus bone turnover. In periodontitis, it can be also released by periodontal ligament fibroblasts, T and B cells under pro-inflammatory stimuli (e.g. TNF-α, IL-1β, and IL-6) in the presence of LPS [21, 26].

**Fig. 1:**
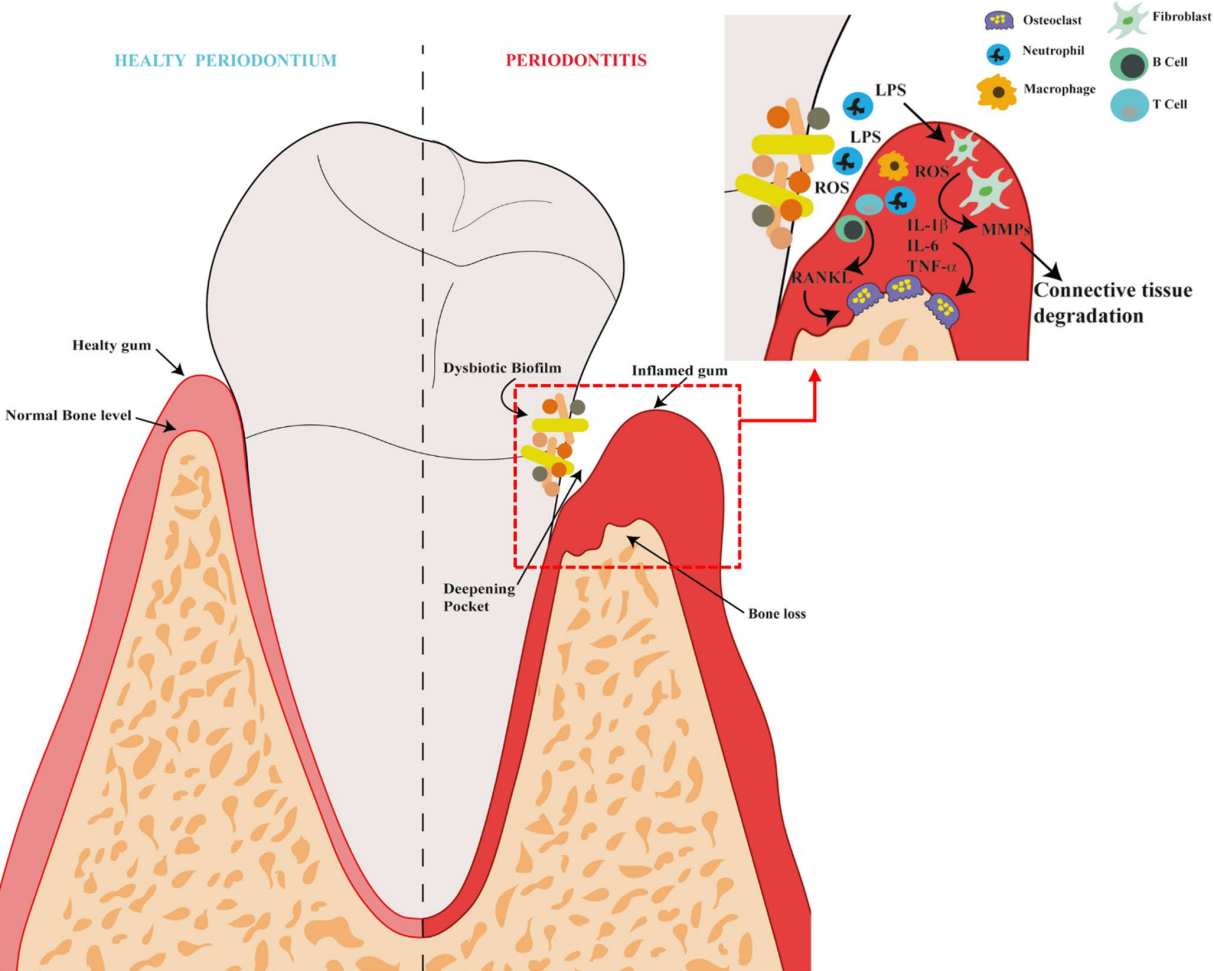
Pathogenesis of periodontitis. Subgingival microbiome dysbiosis increases the population of periodontitis-associated bacteria recruiting inflammatory cells that produce ROS and inflammatory cytokines to counteract the infection. LPS produced by gram-negative bacteria further increases ROS production and inflammation activating nuclear factor kappa-light-chain-enhancer of activated B cells (NF-kB) pathway that increases the secretion of IL-6, IL-1β and TNF-α by T-cells, B-cells, macrophages and neutrophils causing chronic inflammation. The inflammatory environment also increases RANKL expression favoring osteoclastogenesis. Additionally, connective tissue degradation due to increased metalloproteinases (MMPs) expression and alveolar bone resorption by osteoclasts occur

A schematic representation of periodontitis pathogenesis is shown in Fig. 1.

Several studies associated periodontitis to the development of age-related neurodegenerative diseases such as Alzheimer’s and Parkinson’s since the increased cytokines levels caused by gram-negative bacteria present in the periodontium and the bacteria themselves can easily reach the brain due to their anatomical proximity [27], two potential mechanisms by which periodontitis may be involved in the etiology of age-related neurodegenerative diseases have been suggested: (1) migration of bacteria from degenerated periodontal tissue to brain giving rise to a secondary inflammatory reaction and (2) the onset of a systemic chronic inflammation due to periodontitis itself [28]. Since the nuclear factor erythroid 2-related factor 2 (NFE2L2 or NRF2)/ Kelch-like ECH-Associated Protein 1 (KEAP1) (NRF2/KEAP1) signaling pathway is one of the main mechanisms involved in maintaining redox homeostasis and this pathway can be activated by several natural compounds, we were aimed to explore the role of these compounds in activating NRF2/KEAP1 signaling in periodontitis in order to evaluate a potential use of these compounds as supplements for preventing periodontitis or control its progression and, in turn, neurodegenerative diseases such as Alzheimer’s and Parkinson’s diseases.

## Role of periodontitis in neurodegenerative disorders

Continuous evidence showed an association between nervous and stomatognathic system diseases. This association may be due to their anatomical proximity and to the rich circulatory system of the brain and maxillofacial region [29], ii) to the systemic chronic inflammation commonly observed in both dysfunctional systems. The most common age-related neurodegenerative diseases are Alzheimer’s and Parkinson’s diseases. The mechanisms underlying the development of these diseases are not yet known but it is commonly accepted that the initial molecular mechanism that leads to the neuronal degeneration is the chronic neuroinflammation [30]. Periodontitis can play a key role in this process since the increased cytokines levels caused by gram-negative bacteria present in the periodontium can easily reach the brain be due to their anatomical proximity [27].

Patients with severe periodontitis have elevated C-reactive protein levels, a known marker of infection and inflammation [31], compared to unaffected control patients [32]. Moreover, periodontitis leads to systemic and chronic inflammation that can be associated to chronic diseases such as cardiovascular disease, cancers and neurodegenerative disorders [33].

Therefore, there is a direct association between infection in the periodontium and brain damage and an indirect one due to chronic systemic inflammation that is associated with periodontitis and that causes damage at central nervous system level [27, 33–43]. Moreover, Alzheimer’s disease (AD) and Parkinson’s are age-associated diseases characterized by inflammation (inflammaging), as well as periodontitis. Thus, periodontitis aggravates the inflammaging itself.

### Alzheimer’s disease and periodontitis

Alzheimer’s disease (AD) affects memory and cognitive functions and represents about the 80% of all dementia cases in the elderly. Morphologically, AD is characterized by β-amyloid plaques deposition between neurons leading to a progressive synaptic and neuronal loss particularly in the hippocampus and entorhinal cortex, areas designated for learning and memory storage [44]. Moreover, AD is associated with neurofibrillary tangles formation, which are composed of hyperphosphorylated tau protein, that leads to an impairment of physiological functions, apoptosis, and neuronal loss [45]. In addition to amyloid plaques and tau tangles, AD patients also show brain inflammation [27].

Risk factors to develop AD are age, genetics features, diabetes mellitus, stress, and other environmental factors. Moreover, there are many risk factors lifestyle-related such as smoking, mental inactivity, physical inactivity and a poor diet [46].

Recently, it has been proposed that periodontitis would be associated with increased dementia severity and a more rapid cognitive decline in Alzheimer’s disease since periodontitis increases systemic inflammation; therefore, early treatment of periodontitis might be a possible therapeutic strategy for ameliorating the cognitive state of AD patients [40].

Interestingly, a difference in oral microbiomes has been observed between the patients with AD and healthy individuals. Moreover, serum level of anti-*P. gingivalis* LPS antibody titer, a risk factor for AD development [47], was significantly higher in patients with AD [38]. Thus, a modified oral microbiome could cause infection and inflammation, two processes that play a key role in AD pathogenesis.

Additionally, there are emerging evidences that periodontal pathogens could reach the brain since *P. gingivalis* DNA was found in brain tissue from biopsy and in the cerebrospinal fluid of living individuals affected by Alzheimer disease [33]. These findings are interesting since it was demonstrated that LPS from *P. gingivalis* promotes amyloid precursor protein expression in the brain, favoring the deposition of β-amyloid in neurons [43]. In addition to the LPS, the pathogenic potential of *P. gingivalis* is also due to the production of different virulence factors such as gingipains, a group of arginine or lysine specific cysteine proteinase, which are transported to outer bacterial membrane surfaces, and partially released into the extracellular environment [39]. The importance of these virulence factors has been demonstrated in murine models, which revealed that oral administration of gingipain inhibitors blocks gingipain-induced neurodegeneration with consequent reduction of *P. gingivalis* presence in the mouse brain [36]. It has also been demonstrated that peripheral injection of LPS in mouse models induced microglia activation with consequent release of pro-inflammatory cytokines, such as IL-1β, IL-6 and TNF-α promoting a pro-inflammatory environment in the brain and contributing to the exacerbation of cognitive dysfunctions [48, 49]. Increased IL-1β, IL-6, and TNF-α levels were also found in patients with periodontitis and AD suggesting the presence of overlapping mechanisms in the physiopathology of these diseases [27].

It is interestingly to note that an inflammatory diet and low vitamin D levels are associated with periodontitis onset accompanied with a poor cognitive state, highlighting an important role of diet and inflammation in both pathologies [50].

### Parkinson’s disease and periodontitis

Parkinson’s disease (PD) is a neurodegenerative disease caused by an immune system dysfunction and characterized by the loss of dopaminergic neurons within the substantia nigra of the midbrain and cell loss in the other brain areas. The major symptoms are rest tremor, rigidity, and bradykinesia [51]. The risk of developing PD is related to genetic and non-genetic factors, while it is known that age is the leading risk for PD [52]. An epidemiological study reported that individuals with periodontitis had an increased risk of developing PD compared individuals without periodontitis [35]. In addition, it has been reported that dental scaling treatment showed a protective effect against PD onset demonstrating that periodontitis may play a key role in PD onset [34]. Patients with PD have oral health problems, such as painful temporomandibular disorders, caries and periodontal disease [42]. These conditions are maybe due to the PD symptoms since the motor difficulties of PD patients may impair the daily oral hygiene care procedures leading to a poor oral health and periodontal disease. Interestingly, it has been demonstrated that patients with high risk of developing PD present periodontal inflammatory disease. In fact, LPS and the inflammatory cytokines produced during periodontitis may breach the blood brain barrier promoting PD onset and progression [41]. Moreover, it has been demonstrated the presence of gingipain R1 (RgpA), a virulence factor of *P. gingivalis*, in the blood of PD patients suggesting a role of *P. gingivalis* infection in PD onset/progression since it could reduce dopaminergic neurons in the substantia nigra of mice and induce inflammation promoting PD [37].

Overall, it seems that there is a close relationship between periodontitis and neurodegenerative diseases occurrence/progression. In particular, the inflammatory processes and the oral dysbiosis characterizing periodontitis could impair the normal nervous system functions leading to the neurodegenerative diseases previous mentioned. However, there is the possibility that periodontitis could be a comorbidity of patients affected by neurodegenerative diseases.

Thus, an appropriate treatment/prevention of periodontitis could significantly improve the progression/onset of these age-related neurodegenerative diseases.

## NRF2/KEAP1 signaling pathway in periodontitis

**Fig. 2:**
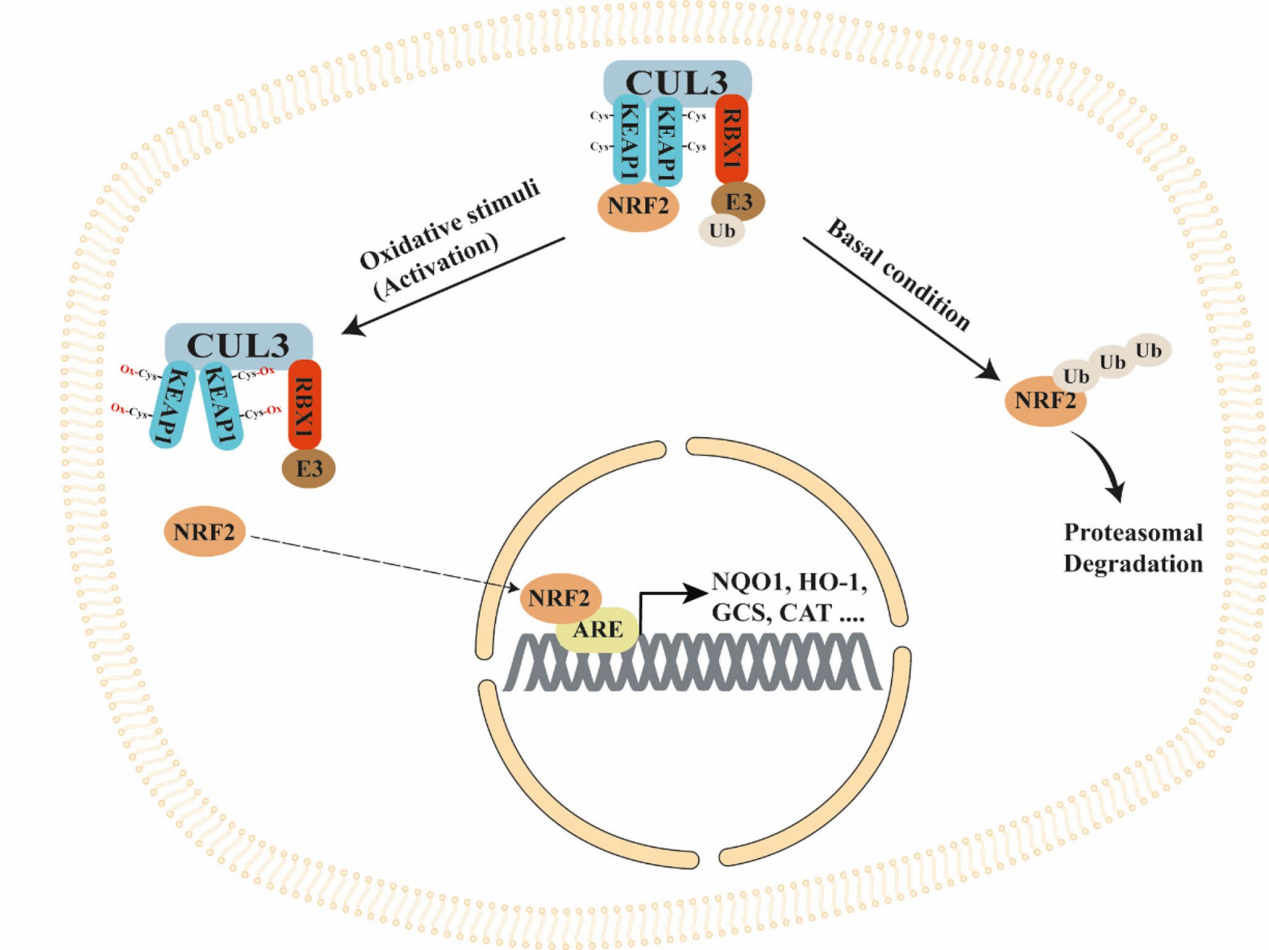
NRF2/KEAP1 signaling pathway regulation. Under basal condition, NRF2 is bound to the KEAP1/CUL3/RBX1 E3-ubiquitin ligase complex that targets NRF2 for proteasomal degradation. However, under oxidative stress, ROS oxidate the cysteine residues of KEAP1 causing a conformational change of KEAP1 that inhibits NRF2 ubiquitination, then leading to NRF2 nuclear translocation and its binding to the ARE regions of antioxidant genes (NQO1, HO-1, etc.)

NRF2 is transcription factor that, under basal conditions, is bound to its repressor KEAP1, which in turn is bound to Cullin 3 (CUL3)/RING-box protein 1 (RBX1)/E3-ubiquitin ligase (KEAP1/CUL3/RBX1) complex [53]. In this form NRF2 is targeted for proteasomal degradation. Under oxidative stimuli, ROS bind the cysteine residues of KEAP1 causing its conformational change that inhibits NRF2 ubiquitination and degradation. In this way, NRF2 migrates into the nucleus and binds antioxidant response elements (AREs) regions present in the promoter of antioxidant genes such as Heme-oxygenase 1 (HO-1) and NAD(P)H: quinone oxidoreductase (NQO1), γ-glutamylcysteine synthetase (GCS) and Catalase (CAT) inducing their expression (Fig. 2) [53].

It has been demonstrated that the NRF2/KEAP1 signaling pathway plays a key role in the onset and progression of several human diseases [54–58] including periodontitis where this pathway modulates redox balance and inflammation of periodontium [59]. In fact, patients with severe periodontitis show a significant decrease of NRF2 expression in gingival tissues [60], favoring the increase of ROS levels and periodontal inflammation [61]. Interestingly, high ROS levels have been detected also in oral cancer suggesting a possible connection between periodontitis and oral cancer development [62–64].

The importance of NRF2/KEAP1 signaling in periodontitis has been further validated in mice model of periodontitis. In fact, NRF2 knockdown in these mice led to an increased oxidative stress in periodontal lesions and alveolar bone loss [65]. Accordingly, overexpression of NRF2 increased NRF2-dependent antioxidant enzymes attenuating oxidative stress-induced apoptosis in human periodontal ligament stem cells (hPDLSCs) [66].

Alterations of NRF2/KEAP1 signaling pathway have also been associated to osteoclastogenesis during periodontitis. In fact, RANKL treatment of mouse primary peritoneal macrophages and RAW 264.7 cells resulted in the up-regulation of KEAP1, ROS production and a decrease of NRF2, HO-1, GCS, and NQO1 expression [67]. These data are in agreement with studies reporting that RANKL induces the nuclear translocation of BTB and CNC homology 1 (Bach1), a repressor of NRF2 [68], causing NRF2 nuclear export and the inhibition of NRF2-dependent antioxidant enzymes transcription, thereby increasing intracellular ROS levels and RANKL-mediated osteoclastogenesis [69, 70]. Similarly, the overexpression of CUL3, an important ubiquitin ligase involved in NRF2 degradation [71], in hPDLSCs treated with LPS from *P. gingivalis* reduced NRF2 levels, cell viability, triggered inflammation and weakened the differentiation and mineralization capabilities of hPDLSCs [72]. Accordingly, NRF2 overexpression up-regulated HO-1, GCS and NQO1 expression, decreased ROS levels while reduced osteoclast differentiation and attenuated bone destruction in both in vitro and in vivo models [67]. NRF2 can also indirectly inhibit osteoclastogenesis reducing IL-6 levels (a promoter of osteoclastogenesis) [73]. Thus, NRF2 activation in osteoclast precursors (or in osteoclast supporting cells) can inhibit osteoclastogenesis and bone destruction decreasing pro-osteoclastogenetic cytokines (e.g. TNF-α, IL-1, and IL-6) and inducing the expression of antioxidant enzymes, thereby attenuating intracellular ROS levels.

Overall, these data suggest that increased NRF2 expression in oral cavity may exert beneficial effects in the prevention/treatment of periodontitis. Furthermore, since periodontitis may be a risk factor for the development of age-related neurodegenerative diseases such as Alzheimer’s and Parkinson’s diseases (see Paragraph 2), strategies aimed at modulating NRF2/KEAP1 signaling during periodontitis may protect against the onset/progression of these neurodegenerative diseases.

## NRF2/KEAP1 signaling activation by natural compounds in periodontitis models

Natural compounds showed important antioxidants and anti-inflammatory effects in several diseases and are widely used as supplements in natural medicine [24, 74, 75]. Therefore, these compounds could be added to chewing sticks, Dentosan-like gels, toothpaste or mouthwash that are normally used as tools for oral hygiene, protecting oral tissues from oxidative stress and inflammation during periodontitis.

### NRF2/KEAP1 signaling activation by natural compounds in periodontitis models

Several studies reported important effects of natural compounds in modulating NRF2/KEAP1 signaling in periodontitis models. We described the molecular effects and characteristics of these compounds in a dedicated review [76]. However, we summarized the main molecular mechanisms of action of these compounds in Table 1. Several of these natural compounds are effective to reduce age-related systemic inflammation in vivo (inflammaging) and senescent associated secretory phenotype in vitro due to cellular aging, i.e. quercetin, curcumin [77, 78].

**Table 1:** NRF2/KEAP1 signaling activation by natural compounds in periodontitis models

Modulator	Model used	Results
Quercetin	H_2_O_2_-exposed hPDLCs, Periodontitis mice model	↑ NRF2, NQO1, CAT and SOD, HO-1 expression, osteogenesis **↓** ROS, DNA damage, cellular senescence alveolar bone loss
Biochanin A	Periodontitis rat model	↓ alveolar bone resorption, IL-1β, TNF-α, ROS levels ↑ NRF2 expression
Curcumin	hPDLSCs	↑ AKT phosphorylation, NRF2 expression, osteogenesis
	*F. nucleatum*-exposed H400 cell line	↓ NF-κB nuclear translocation, IL-1β, TNF-α and IL-8 ↑ NRF2 and HO-1 expression.
10-oxo-trans-11-octadecenoic acid	GECs	↑ NRF2, HO-1 and NQO1 expression ↓ ROS levels.
Caffeic acid phenethyl ester	Primary murine macrophages, RAW264.7 cells, Primary human gingival fibroblasts	↑ HO-1 expression ↓ IL-1α and IL-1β levels.
Paeonol	Periodontitis rat model	↓ RANKL, osteoclastogenesis, NF-κB activation, IL-1β, IL-6, TNF-α and ROS levels ↑ NRF2 and HO-1 expression, GSH levels
Euphorbia factor L1	Mice bone marrow-derived macrophages	↓osteoclastogenesis, bone resorption, NF-κB activation, ROS levels ↑NRF2, SRX, PRXs and TRXs expression.
Resveratrol	Periodontitis rat model	↓ alveolar bone loss, TNF-α, IL-1, IL-6 levels, NF-κB activation ↑ NRF2 expression
	Periodontitis mouse model.	↓ alveolar bone loss, ROS ↑ NRF2 expression
Sulforaphane	Differentiated HL60 cells (as a neutrophil model) Primary neutrophils from patients.	↑ intracellular GSH/GSSG ratio, NRF2, NQO1, GCLC and GCLM expression
	GECs	↑ NRF2 and HO-1 expression
**NRF2 activation on RANKL-induced osteoclastogenesis in periodontitis**		
Dehydrocostus lactone	RANKL-stimulated RAW264.7 cells	↓ NR-κB activation, ROS levels, osteoclastogenesis ↑ NRF2, NQO1, sulfiredoxin (SRX) and peroxiredoxin-1 (PRX1) expression
Hesperetin	RAW264.7 cells	↓osteoclastogenesis, NR-κB activation, ROS levels ↑ NRF2, HO-1 and NQO1 expression
**NRF2 modulators in LPS-exposed models of periodontitis**		
Notopterol	LPS-stimulated HGFs	↓ IL-1β, IL-32, and IL-8 levels, NR-κB activation, ROS levels ↑ NRF2, HO-1, CAT, and NQO1 expression HO-1, NQO1
Isorhamnetin	LPS-stimulated HGFs	↓ IL-6 and IL-8 levels, NR-κB activation ↑ NRF2, HO-1 expression
Magnolol	LPS-stimulated RAW 264.7	↓ TNF-α and IL-1β levels, NF-κB activation ↑ NRF2 and HO-1 expression
Resveratrol	LPS-stimulated HGFs and Rats with periodontitis	↓ROS levels, alveolar bone loss, osteoclastogenesis ↑ NRF2 and HO-1 expression
	LPS-stimulated hPDLSCs	↓ IL-1β and IL-6 levels, NR-κB activation ↑ NRF2 and HO-1 expression, osteogenesis
Lindenenyl acetate	LPS-stimulated hPDLCs	↓TNF-α, IL-1β, IL-6 and IL-12 levels ↑NRF2 nuclear translocation, HO-1 expression and activity
Schisandrin	LPS-stimulated RAW 264.7	↓TNF-α, IL-1β and IL-6 levels, NF-kB activation ↑ NRF2 and HO-1 expression
Sappanchalcone	LPS-stimulated HDPCs and hPDLCs	↓ IL-1β, TNF-α, IL-6 and IL-12 levels ↑NRF2 and HO-1 expression
**NRF2 as anti-pyroptotic target in periodontitis**		
Kynurenic acid	LPS‑induced THP‑1 macrophage	↓ pyroptosis, IL-1β, IL-18 and TNF-α levels, ROS levels ↑ NRF2 and HO-1 expression
Epigallocatechin-3-gallate	Periodontitis rat model	↓ alveolar bone loss, IL-1β, IL-18, TNF-α levels, pyroptosis, NF-κB activation, ROS levels ↑ NRF2 and HO-1 expression
Silibinin	Periodontitis rat model	↓ alveolar bone loss, ROS levels, NF-κB activation, TNF-α, IL-1β, and IL-6 levels, pyroptosis ↑ NRF2 expression
Chlorogenic acid	LPS-induced HGFs	↓ IL-1β, IL-18 levels, ROS levels ↑ NRF2 and HO-1 expression
	LPS-induced IHOKs	↓ ROS levels, NF-κB activation, ↑ NRF2 translocation and HO-1 expression

↑ (increased); ↓ (decreased); hPDLCs (human periodontal ligament cells); HGFs (human gingival fibroblasts); HDPCs (human dental pulp); hPDLSCs (human periodontal ligament stem cells); GECs (gingival epithelial cells); IHOKs (immortalized human oral keratinocytes)

### NRF2/KEAP1 signaling activation by natural extract in periodontitis models

In addition to the natural compounds discussed in the previous paragraph, NRF2/KEAP1 signaling can be modulated by natural extracts, which contain more than one active compound.

Edible mushrooms are a good source of anti-inflammatory and antioxidant substances, and may play an important role in counteracting inflammation and oxidative stress [79, 80]. *Agaricus blazei* Murrill polysaccharide (ABMP) is a natural compound present in the edible mushroom species *A. blazei* Murrill and showed important anti-inflammatory effects [81]. It has been reported that ABMP increased NRF2 expression, alleviated gingival recession, reduced IL-1𝛽 and TNF-𝛼 levels, alveolar bone resorption, and osteoclasts number in periodontitis rat model. Moreover, ABMP induced autophagy, a cellular process that favors cell survival during stress conditions, in periodontitis rat model. Interesting, inhibition of NRF2 with its synthetic inhibitor ML385 in LPS-treated human periodontal ligament cells (hPDLCs) reversed the beneficial effects of ABMP leading to increased IL-1𝛽 levels and reduced autophagy demonstrating that ABMP induces autophagy in hPDLCs through the upregulation of NRF2 [82].

Ginseng (*Panax ginseng* C.A. Meyer) is a medicinal plant widely used in Korea, Japan, Russia and China for the treatment of cardiovascular diseases. The main Ginseng active compound is the ginsenoside Rb1, which showed important antioxidant and anti-inflammatory activities [83]. *Panax ginseng* fruit extract (PGFE) treatment suppressed TNF-α, IL-1β and IL-6 levels while increased NRF2 and HO-1 expression in hPDLCs exposed to LPS from *P. gingivalis*. Importantly, PGFE induced osteogenesis in LPS-stimulated cells. Notably, inhibition of HO-1 with tin protoporphyrin IX (SnPP) (a specific HO-1 inhibitor) increased the inflammatory cytokines and inhibited osteogenesis in LPS-exposed cells. It was also demonstrated that PGFE was able to reduce periodontal inflammation, increase alveolar bone volume and bone mineral density in periodontitis mouse model [84].

It has been widely demonstrated that edible marine algae can be an important source of natural antioxidant compounds protecting cells from oxidative stress [85–87]. *Ecklonia cava* is an edible marine brown alga known to be a rich source of phlorotannins, which have an important antioxidant activity [88]. An interesting study found that *Ecklonia cava* ethanol extract (ECE) treatment of RAW 264.7 cells exposed to LPS from *P. gingivalis* significantly decreased TNF-α, IL-1β and IL-6 gene expression. Moreover, ECE treatment upregulated NRF2 and HO-1 expression while decreased NF-κB activation. ECE also reduced tooth mobility, prevented alveolar bone loss, decreased inflammatory cell infiltration, IL-1β production, and MMP-2/-9 expression in gingival tissues of periodontitis rats model demonstrating that ECE has important therapeutic effects for the alleviation of periodontal disease [89].

Several shrubs contain polyphenols, flavonoids and other bioactive compounds in their fruits/leaves with important antioxidant, antitumor, and anti-inflammatory functions [90, 91]. *Osmanthus fragrans* is an evergreen shrub with antioxidant, antitumor and anti-inflammatory effects [92]. It has been reported that treatment of hPDLCs cells with extract of *Osmanthus fragrans* (EOF) significantly inhibited the growth and the collagenase activity of *P. gingivalis*. Moreover, EOF significantly inhibited the *P. gingivalis* LPS-stimulated production of IL-6 and IL-8, and upregulated the expression NRF2, HO-1 and NQO1 demonstrating important antioxidant and anti-inflammatory effects [93]. Another important shrub with beneficial properties is *Acanthopanax senticosus* (AS), also known as Siberian ginseng, a traditional medicinal plant used in East Asia for its anticancer, anti-diabetes, and anti-inflammatory properties [94]. Kim and colleagues studied the anti-inflammatory activity of AS extract (ASE) in macrophage RAW 264.7 cell line stimulated with LPS from *P. gingivalis* and found that ASE significantly induced the expression and activity of HO-1 upregulating NRF2 expression. Moreover, ASE suppressed the production of TNF-α, IL-1β, and IL-6, and decreased the nuclear translocation of activator protein-1 (AP-1) and NF-κB by inhibiting the phosphorylation of IκB-α. Furthermore, ASE treatment inhibited signal transducer and activator of transcription (STAT)1 phosphorylation while activated STAT3 phosphorylation. Thus, ASE exerts its anti-inflammatory effects on *P. gingivalis* LPS-stimulated macrophages decreasing AP-1 and NF-κB activity, modulating STAT1 and STAT3 phosphorylation, and upregulating NRF2 and MAPK signaling pathways [95].

Important antioxidant and anti-inflammatory compounds have been found in several medical herb [96]. *Bambusae Caulis in Taeniam* (BC) is a medicinal herb used in China to treat fever, diarrhea, and inflammation [97]. Jin and colleagues investigated the role of BC extract (BCE) on RAW264.7 macrophages stimulated with LPS from *P. gingivalis* and found that BCE treatment inhibited the production of TNF-α, IL-1β, and IL-6, and suppressed the nuclear translocation of NF-κB. Moreover, BCE increased the nuclear translocation of NRF2 and HO-1 expression. Importantly, HO-1 inhibition with SnPP attenuated BCE’s anti-inflammatory effects. Finally, inhibition of MAPK signaling pathway (using the p38 inhibitor SB203580) reduced BCE-induced HO-1 expression. Thus, the anti-inflammatory effects of BCE are due to the activation of NRF2/HO-1 axis and mitogen-activated protein kinase (MAPK)/NF-κB signaling [98]. Another important medical plant is *Ginkgo biloba*, a herb widely used in oriental traditional medicine due to the important anti-inflammatory, antioxidant and anticancer effects of its leaves and seeds [99]. Ryu and colleagues showed that *G. biloba* extracts exhibited anti-inflammatory activities, downregulated MAPK (ERK, JNK, and p38) signaling pathways, and increased NRF2 and HO-1 expression in LPS-treated RAW 264.7 cells. HO-1 inhibition with SnPP reduced the secretion pro-inflammatory cytokines (TNF-α, IL-1β and IL-6) suggesting that the anti-inflammatory effects of *G. biloba* extract are due to the activation of NRF2/HO-1 axis in RAW 264.7 cells exposed to *P. gingivalis* LPS [100].

Bioactive compounds can also be found in the fruits of several trees [101]. *Cratoxylum cochinchinensis* is a tree widely distributed in several Southeast Asian countries. The xanthone compounds isolated from the fruit of this tree showed important antioxidant, antibacterial and anti-inflammatory effects [102]. Ruangsawasdi and colleagues showed that xanthone extracts isolated from the young fruit of *Cratoxylum cochinchinensis* reduced ROS levels inducing NRF2 and HO-1 expression in hPDLSCs. Moreover, the xanthone extracts potentiated hPDLSCs osteogenic differentiation under oxidative stress [103].

Beneficial anti-inflammatory and antioxidant effects have also been found in the leaves extract of several plants [104]. *Ixeris dentata* (IXD) is a flowering plant with anti-inflammatory effects [105] and it has been reported that IXD leaves extract and *Lactobacillus gasseri* alone or in combination inhibit alveolar bone loss, favored osteogenesis, increased NRF2 and HO-1 expression, and reduced ROS, TNF-α, IL-1β and IL-6 levels in periodontitis mouse model. Moreover, IXD and *Lactobacillus gasseri* alone or in combination reduced NF-kB activation. The osteogenic capacity of IXD and *Lactobacillus gasseri* was also demonstrated in RANKL-induced osteoclast formation of RAW264.7 cells. However, it deserves to be mentioned that the effects of IXD and *Lactobacillus gasseri* were way stronger if used as cotreatment [106].

The studies discussed in this section are summarized in Table 2.

**Table 2:** NRF2/KEAP1 signaling activation by natural extracts in periodontitis models

Modulator	Model used	Results	Ref.
*Agaricus blazei* Murrill polysaccharide (ABMP)	LPS-stimulated hPDLCs and periodontitis rat model	↓ gingival recession, reduced IL-1𝛽 and TNF-𝛼 alveolar bone resorption, osteoclasts number ↑ NRF2 expression, autophagy	[82]
Panax ginseng fruit extract (PGFE)	LPS-stimulated hPDLCs and periodontitis mice model	↓ TNF-α, IL-1β and IL-6, periodontal inflammation ↑ NRF2 and HO-1 expression, osteogenesis, alveolar bone volume and bone mineral density	[84]
Ecklonia cava ethanol extract (ECE)	LPS-stimulated RAW 264.7 cell line and periodontitis rat model	↓ NF-κB activation, TNF-α, IL-1β and IL-6, tooth mobility, alveolar bone loss, inflammatory cell infiltration, MMP-2/-9 expression ↑NRF2 and HO-1 expression	[89]
Extract of *Osmanthus fragrans* (EOF)	LPS-stimulated hPDLCs	↓ growth and the collagenase activity of *P. gingivalis*, IL-6 and IL-8 levels ↑ NRF2, HO-1 and NQO1 expression	[93]
*Bambusae Caulis in Taeniam* extract (BCE)	LPS-stimulated RAW 264.7 cell line	↓ TNF-α, IL-1β, and IL-6, NF-κB activation ↑ NRF2 and HO-1 expression	[98]
*Acanthopanax senticosus* extract (ASE)	LPS-stimulated RAW 264.7 cell line	↓ TNF-α, IL-1β, and IL-6 levels, NF-κB activation (inhibiting IκB-α phosphorylation), STAT1 phosphorylation ↑ NRF2 and HO-1 expression, ERK and JNK signaling activation, STAT3 phosphorylation.	[95]
*Ginkgo biloba* extract	LPS-stimulated RAW 264.7 cells	↓ TNF-α, IL-1β and IL-6 levels, MAPK signaling activation ↑ NRF2 and HO-1 expression	[100]
Xanthone extracts from the young fruit of *Cratoxylum cochinchinensis*	hPDLSCs	↓ ROS levels ↑ NRF2 and HO-1 expression, osteogenic differentiation	[103]
*Lactobacillus gasseri* and *Ixeris dentata* (IXD) extracts	periodontitis rat model and RAW264.7 cells	↓ alveolar bone loss, ROS, NF-kB activation, TNF-α, IL-1β and IL-6 levels ↑ osteogenesis, NRF2 and HO-1 expression	[106]

↑ (increased); ↓ (decreased); hPDLCs (human periodontal ligament cells); human periodontal ligament stem cells (hPDLSCs)

## NRF2/KEAP1 signaling in periodontitis complicated by diabetes

Diabetes mellitus is a metabolic disorder characterized by insulin deficiency or resistance which leads to a chronic hyperglycemia that damage several organs and tissues causing important comorbidities [107]. Diabetes mellitus is an important risk factor for periodontitis occurrence and progression since it significantly increases periodontitis occurrence and progression [108]. Although the role of diabetes mellitus in periodontitis is still not fully understood, inflammation and oxidative stress related to chronic hyperglycemia play a key role in the onset and progression of this disease [109].

In our previous review [76], we highlighted the beneficial effects of baicalein, a flavonoid found in *Scutellaria baicalensis* Georgi, and Magnolol, a natural compound found in *Magnolia officinalis*. In fact, these compounds significantly reduced ROS levels and inflammation in human gingival epithelial cells (hGECs) and human gingival fibroblasts (HGFs) exposed to high glucose levels and advanced glycation end-products (AGEs), respectively. In both these studies it was found that the beneficial effects of these compounds were due to an increase in the NRF2 and NRF2-depending antioxidant genes expression [76].

In addition to baicalein and magnolol, interesting results were also obtained by *albiflorin*, a monoterpene glycoside compound derived from the root and stem of the *Paeonia lactiflora*. In fact, albiflorin treatment of AGEs-exposed HGFs reduced ROS levels and enhanced cell viability. Albiflorin also decreased IL-6 and IL-8 levels, as well as receptor for advanced glycation end-products (RAGE) and matrix metallopeptidase (MMP)-1 expression. Authors also demonstrated that the anti-inflammatory effects of albiflorin were due to the inhibition of the NF-κB pathway and the activation of NRF2 pathway [110].

In addition to the natural compounds previously mentioned, fungal immunomodulatory proteins (FIPs) from *Ganoderma microsporum* showed important anti-inflammatory effects in HGFs exposed to AGEs and LPS from *P. gingivalis.* In fact, FIPs reduced ROS production and cell senescence while increased NRF2 and HO-1 expression. FIPs also decreased NF-κB phosphorylation reducing IL-6 and IL-8 levels [111].

Patients with diabetes mellitus can also be affected by dysbiosis, which is also the basis of periodontitis [112, 113]. It has been reported that the probiotic *Clostridium butyricum* MIYAIRI 588 (CBM588) is an effective therapeutic approach for gut dysbiosis [114]. Interestingly, intragastric supplementation with CBM588 in diabetes mellitus-induced periodontitis-induced diabetic mouse model, alleviated periodontal bone destruction and reduced oxidative stress increasing 4-hydroxybenzenemethanol (4-HBA) in serum. It was also demonstrated that 4-HBA promoted NRF2 activation and ameliorated inflammatory bone destruction in patients affected by periodontitis and diabetes mellitus. Thus, CBM588 exerts important protective effects in both diabetes mellitus and diabetes mellitus-associated to periodontitis regulating gut microbiota and alleviating oxidative damage [115].

## NRF2 as anti-ferroptosis target in periodontitis

Ferroptosis is a type of regulated cell death process driven by iron-dependent lipid peroxidation, ROS and cell metabolism; it is involved in several diseases including cancer, inflammatory diseases [116] and periodontitis [117]. NRF2 has been proposed as an anti-ferroptosis target in several diseases since it can regulate redox state of the cells [118, 119].

NRF2 and glutathione peroxidase 4 (GPX4) expression are lower in periodontal tissues of periodontitis mice model. In mice with periodontitis, ferroptosis induction (by using erastin) aggravated alveolar bone loss and further decreased NRF2, GPX4 expression and GSH content inducing ROS formation and inhibiting osteogenic differentiation of primary mouse mandibular osteoblasts. Importantly, silencing of NRF2 enhanced the effects previously mentioned demonstrating that NRF2 plays an essential role in regulating ferroptosis and differentiation of osteoblasts [120].

Another study found that macrophage cell line RAW 264.7 exposed to LPS undergone ferroptosis and showed lower expression of NRF2. Accordingly, overexpression of NRF2 reduced ROS levels and ferroptosis by upregulating ferroptosis suppressor protein 1 (FSP1) expression [121]. Additionally, inhibition of ferroptosis (by using ferrostatin-1) significantly decreased TNF-α, Il-1β, Il-6 levels. Interesting, in vitro co-culture experiments showed that the osteogenic potential of mouse bone marrow stromal cells (BMSCs) was decreased by TNF-α produced by LPS-exposed RAW 264.7 macrophages undergoing ferroptosis. Thus, in periodontitis there could be an autocrine-paracrine loop in ferroptotic macrophages that can inhibit osteogenesis through the NRF2/FSP1/ROS pathway resulting in bone loss [122]. The role TNF-α in ferroptosis and inflammation was also demonstrated by Wu and colleagues. In fact, TNF-α treatment of hPDLSCs increased ferroptosis, and IL-6 and IL-8 levels but these effects were reversed by stimulating NRF2 activation with bomidin, a new recombinant antimicrobial peptide (AMP) [123].

Thus, NRF2 activation in periodontitis may significantly contribute to the suppression of ferroptosis alleviating inflammation and alveolar bone loss.

## Conclusions and future perspectives

Periodontitis is a frequent disease caused by the migration of anaerobic gram-negative bacteria into the subgingival space leading to persistent inflammation, periodontal damage and alveolar bone loss. Neutrophils and LPS are the major causes of increased ROS levels during periodontitis since neutrophils use ROS to fight the infection and LPS is released from gram negative bacteria present in the periodontal biofilm. The increased amount of ROS and LPS during periodontitis also favors the production of pro-inflammatory mediators by inflammatory cells and periodontal tissue causing periodontal inflammation and favoring alveolar bone loss due to an excessive osteoclast formation and activation [13, 18–20] but also a chronic systemic inflammation which can be associated to an increase risk to develop diseases such as AD and Parkinson. It deserves to be pointed out that, although cytokines elevation and bacterial components such as LPS have been discussed as possible mediators in AD and Parkinson occurrence/progression, none of them have been firmly established as causal factors.

The NRF2/KEAP1 signaling pathway plays a key role in the pathogenesis of periodontitis since it regulates redox balance and inflammation of periodontium. The importance of this signaling in periodontitis is demonstrated by the fact that NRF2 expression is decreased in gingival tissues of severe periodontitis patients while ROS levels and periodontal inflammation are increased. These data were further confirmed by NRF2 knockdown in mice model of periodontitis, showing a more severe alveolar bone loss and an increased oxidative stress in periodontal lesions [59–61, 65]. Therefore, treatments modulating this pathway may be useful to reduce periodontitis and potentially associated diseases such as AD and Parkinson. In fact, the studies discussed in this review highlighted that the activation of NRF2/KEAP1 signaling by natural compounds is often accompanied by the inhibition of NF-κB signaling, leading to decreased TNF-α, IL-1β, IL-6, IL-32, and IL-8 levels. Moreover, the natural compounds activated the NRF2 pathway upregulating HO-1, NQO1, and CAT expression, thereby decreasing ROS levels. These treatments also reduced the expression of MMP-2, MMP-9, and the activation of MAPK signaling pathway. Looking at the studies discussed in paragraph 5, it came out that natural compounds such as baicalein, magnolol, albiflorin, fungal immunomodulatory proteins (FIPs) from *Ganoderma microsporum* and probiotic (*Clostridium butyricum* MIYAIRI 588) can activate NRF2/KEAP1 signaling in in vitro and in vivo models of diabetes-associated periodontitis reducing inflammation, ROS levels and alveolar bone loss while favoring osteoblast differentiation. Saliva is an interesting diagnostic body fluid since it can allow a non-invasive detection of potential diagnostics markers. In fact, alterations of oral health such as those characterizing periodontitis significantly alter saliva’s composition. As discussed in this review, periodontitis is associated with oxidative stress and the latter can be detected with specific markers detectable in the saliva. Important oxidative stress markers are: Advanced oxidation protein products (AOPP), Thiobarbituric acid reactive substances (TBARS), 8-hydroxyguanosine (8-OHdG) but also others [124]. A “smart” toothbrush embedded with built-in sensors able to quantify one or more of these oxidative stress markers during the routing oral hygiene may detect increased oxidative stress levels in oral cavity allowing an early detection of oral diseases such as periodontitis. These alterations could be communicated directly to the dentist and/or to the general practitioner through the connection of the “smart” toothbrush to the internet. The dentist and/or the general practitioner can call the patients for a check and evaluate the prescription of a supplement based on one or more of the compounds discussed in this review. Thus, oral hygiene control to prevent periodontitis or keep it under control using ad hoc tools combined to the use of oral supplements to reduce the local and therefore systemic inflammatory state may significantly reduce the inflammatory effects on the central nervous system preventing/ameliorating Alzheimer’s and Parkinson’s diseases occurrence/progression.

## Data Availability

No datasets were generated or analysed during the current study.

## Abbreviations

AD: Alzheimer’s disease
ABMP: Agaricus blazei Murrill polysaccharide
AOPP: Advanced oxidation protein products
AP-1: activator protein 1
Bach1: BTB and CNC homology 1
BMSCs: bone marrow stromal cells
CAL: clinical attachment loss
AGEs: advanced glycation end-products
AMP: antimicrobial peptide
ARE: antioxidant response element
ASE: Acanthopanax senticosus extract
BCE: Bambusae Caulis in Taeniam extract
CAT: catalase
CUL3: cullin 3
ECE: Ecklonia cava ethanol extract
EOF: Osmanthus fragrans extract
FIPs: fungal immunomodulatory proteins
FSP1: ferroptosis suppressor protein 1
GPX4: glutathione peroxidase 4
GSH: glutathione
GCS: γ-glutamylcysteine synthetase
HO-1: heme oxygenase 1
hGECs: human gingival epithelial cells
HGFs: human gingival fibroblasts
hPDLCs: human periodontal ligament cells
hPDLSCs: human periodontal ligament stem cells
IL: interleukin
IXD: Ixeris dentata
KEAP1: Kelch Like ECH Associated Protein 1
LPS: lipopolysaccharide
MAPK: mitogen-activated protein kinase
MMP: matrix metallopeptidase
NF-κB: nuclear factor kappa-light-chain-enhancer of activated B cells
NQO1: NAD(P)H quinone dehydrogenase 1
NRF2: nuclear factor erythroid 2-related factor 2
PD: Parkinson’s disease
PGFE: Panax ginseng fruit extract
RAGE: receptor for advanced glycation end-products
RANKL: receptor activator of nuclear factor-κB ligand
RBX1: RING-box protein 1
RgpA: gingipain R1
ROS: reactive oxygen species
SnPP: tin protoporphyrin IX
STAT: signal transducer and activator of transcription
TBARS: Thiobarbituric acid reactive substances
TNF: tumor necrosis factor
4-HBA: 4-hydroxybenzenemethanol
8-OHdG: 8-hydroxyguanosine
